# Supplementary material for: Using safe and ethical technology to prevent and respond to sexual and interpersonal violence during adolescence and young adulthood: Identifying evidence, best practices, and pathways forward—A global scoping review protocol
Source: PLoS One. 2025 Aug 13;20(8):e0320709. doi: 10.1371/journal.pone.0320709 (PMC12349042; doi:10.1371/journal.pone.0320709)
Supplement: S2 Table — (DOCX) [file pone.0320709.s002.docx]

S2 Table: Search Strategy for Included Databases in Scoping Review

(Limited to Date Range: 2008–2025 and English Language)

| **#** | **Concept** | **Result** |
| --- | --- | --- |
| **PubMed(**Medline is part of PubMed) | | |
| #1 | Intimate Partner Violence[mesh] OR Gender-Based Violence[mesh] OR Interpersonal Violence[mesh]OR Sex Offenses[mesh] OR Sexual Harassment [mesh] OR Gender Based Violence[tiab] OR violence against women[tiab] OR Intimate Partner Violence[tiab] OR Intimate Partner Abuse[tiab] OR Interpersonal Violence[tiab] OR Sexual Abuse[tiab] OR Sexual Violence[tiab] OR Sexual Assault[tiab] OR femicide[tiab] OR Sexual Harassment[tiab] | 81,855 articles |
| #2 | Telemedicine[mesh] OR Mobile Applications[mesh] OR Digital Health[mesh] OR Digital Health Technolog*[tiab] OR Mobile Application*[tiab] OR Mobile App[tiab] OR Mobile Apps[tiab] OR Smartphone Apps[tiab] OR Smartphone App[tiab] OR Portable Electronic App[tiab] OR Portable Electronic Application*[tiab] OR Portable Software App[tiab] OR Portable Software Application*[tiab] OR mobile health[tiab] OR ehealth[tiab] | 70,471 articles |
|  | #1 AND #2 | 288  articles |
| **Embase** | | |
| #1 | 'partner violence'/exp OR 'gender based violence'/exp OR 'femicide'/exp OR 'interpersonal violence'/exp OR 'sexual harassment'/exp ('gender based violence' OR ‘violence against women’ OR 'intimate partner violence' OR 'partner abuse' OR 'spouse abuse' OR 'partner violence' OR 'femicide' OR 'interpersonal violence'):ab,ti,kw | 13,793  articles |
| #2 | 'telemedicine'/exp OR 'mobile application'/exp OR 'digital health'/exp OR 'digital health technology'/exp (‘Mobile Application*’ OR ‘Mobile App’ OR ‘Mobile Apps’ OR ‘Smartphone Apps’ OR ‘Smartphone App’ OR ‘Portable Electronic App’ OR ‘Portable Electronic Application*’ OR ‘Portable Software App’ OR ‘Portable Software Application*’ OR ‘mobile health’ OR ‘ehealth’):ab,ti,kw | 21,910  articles |
|  | #1 AND #2 | 19  articles |
| **Scopus** | | |
| #1 | “gender based violence” OR “violence against women” OR “intimate partner violence” OR “partner abuse” OR “spouse abuse” OR “partner violence” OR “femicide” OR “interpersonal violence” OR “sexual harassment” | 53,944 articles |
| #2 | “Mobile Application*” OR “Mobile App” OR “Mobile Apps” OR “Smartphone Apps” OR “Smartphone App” OR “Portable Electronic App” OR “Portable Electronic Application*” OR “Portable Software App” OR “Portable Software Application*” OR “mobile health” OR “ehealth” OR “digital health” OR “digital health technology” | 123,681 articles |
|  | #1 AND #2 | 156 articles |
| **Web of Science** | | |
| #1 | “gender based violence” OR “violence against women” OR “intimate partner violence” OR “partner abuse” OR “spouse abuse” OR “partner violence” OR “femicide” OR “interpersonal violence” OR “sexual harassment” | 42,493 articles |
| #2 | “Mobile Application*” OR “Mobile App” OR “Mobile Apps” OR “Smartphone Apps” OR “Smartphone App” OR “Portable Electronic App” OR “Portable Electronic Application*” OR “Portable Software App” OR “Portable Software Application*” OR “mobile health” OR “ehealth” OR “digital health” OR “digital health technology” | 77,486 articles |
|  | #1 AND #2 | 113 articles |
