# Supplementary material for: Using safe and ethical technology to prevent and respond to sexual and interpersonal violence during adolescence and young adulthood: Identifying evidence, best practices, and pathways forward—A global scoping review protocol
Source: PLoS One. 2025 Aug 13;20(8):e0320709. doi: 10.1371/journal.pone.0320709 (PMC12349042; doi:10.1371/journal.pone.0320709)
Supplement: S3 Table — (DOCX) [file pone.0320709.s003.docx]

**S3 Table. Data extraction form**

| **Scoping Review Details** | |
| --- | --- |
| **Scoping Review title**: |  |
| **Review objective/s**: |  |
| **Review question/s:** |  |
| **Evidence source Details and Characteristics** | |
| Authors |  |
| Date of study |  |
| Title of study |  |
| Study objective/aims |  |
| Types of evidence source (cross-sectional, prospective cohort, retrospective cohort, qualitative, case-control, randomized control trial) |  |
| Type of study (e.g, primary research, and secondary analysis) |  |
| Study design |  |
| Country/region that study has been conducted |  |
| Age of participants |  |
| Sex of participants |  |
| Gender of participants |  |
| Number of participants |  |
| Details of data collection (e.g. face-to-face interview, online survey etc.) |  |
| What data analysis method was used? Provide description if necessary. |  |
| **Details/Results extracted from source of evidence** | |
| What types of technology have been utilized to address GBV? (e.g., mobile apps, websites, software) |  |
| Has technology been effective in facilitating incident reporting? |  |
| Has technology supported survivors in ensuring safety and accessing support? |  |
| What ethical considerations arise when using technology to report GBV (e.g., privacy, traceability, consent requirements, data storage, and data protection)? |  |
| How have these ethical issues been addressed? |  |
| Recommendations for future research, policy, or practice |  |
| Limitations of study |  |
| Other key findings relevant to research questions |  |
