# Supplementary material for: Using safe and ethical technology to prevent and respond to sexual and interpersonal violence during adolescence and young adulthood: Identifying evidence, best practices, and pathways forward—A global scoping review protocol
Source: PLoS One. 2025 Aug 13;20(8):e0320709. doi: 10.1371/journal.pone.0320709 (PMC12349042; doi:10.1371/journal.pone.0320709)
Supplement: Attachment — Submitted filename: PONE-D-25-09607_6.5.2025_Peer Review.docx [file pone.0320709.s004.docx]

Please accept my review of manuscript #PONE-D-25-09607, which is being considered for publication in PLOS ONE. This manuscript outlines an evidence-based protocol with which the authors plan to conduct a scoping review. The focus of this review will be technology’s impacts on either mitigating or facilitating sexual and interpersonal violence (SIV) amongst 8-25 year olds. The protocol has many strengths, including filling a needed gap in the literature on factors influencing technology-facilitated or -mitigated SIV, responding to a rising form of SIV with both a preventative and responsive intention, as well as the rigor and depth of the proposed scoping review. In addition to these strengths, I included suggestions for improvements below, which focus on increasing clarity of wording and purpose, flow, citations, and minor suggestions on methodology. Overall, I think this is an important study and a well-devised protocol that will be ready for publication with some revisions.

**Paper-wide suggestions:**

- Please use consistent either APA or AMA-style in-text citation formatting. AMA would include superscript numbering, while APA would include the author last names and publication date in parentheses.
- General recommendation: please clarify more thoroughly, in both the introduction and method, why a scoping review is the best choice of review type for this study.

**Abstract:**

- Please include the full version of the abbreviation PRISMA-P.
- Briefly mention that Covidence is a type of software for those who are unfamiliar.

**Introduction:**

- I have an issue with the way SIV is presented in the first paragraph. Think about and revisit.
- Line 67: “age-groups” does not need a hyphen.
- Line 70: “(11,12**).S**exual”: insert a space.
- Line 80: “Adolescents and young adults are easy **target** of “: change to targets.
- Line 81: “both in partner and non-partner contexts”: please change to “partnered and non-partnered”
- Line 82: the word “penetration” is inappropriately used in this sentence. Do you mean frequency or prevalence?
- Line 85: please say “in **the** United States”
- Line 85: “Youth aged 13-17 years were found to be at the highest risk of technology-assited abuse, with majority of the perpetrators being older than this age-group (18).”
  - Perhaps a more precise and grammatically correct way to word this is something like: In fact, one study found that youth aged 13-17 were the age group with the highest risk of being victimized by technology-assisted abuse, with the majority of perpetrators being older than this age group (18).
- Line 94: “use(21,22)” insert a space after “use.”
- Lines 94-98: Please be more specific about the “topics” that adolescents may develop “incorrect” perceptions about; also, please be more specific about what is meant by “content-based data practices” and survivor-centered “design.” I also suggest citing all of these claims.
- Line 103: what is an example of a potential ethical issue that may have been reported in the past?
- Lines 113-120: please be sure to cite all claims.

**Method:**

- General recommendation: please include a description of how the “needs of diverse populations” will be methodologically approached, such as whether it will be a specific research question or piece of extracted data, as this goal is mentioned in both the introduction and discussion.
- Line 135: add period.
- Research question 2: This is a great question, yet it might be more scientifically fruitful to answer if it is reworded as an open question. For example, “*To what extent* has technology been effective in facilitating…” may give you more room to answer this question qualitatively.
- The parenthetical portion of your 3rd research question is important background information about ethical considerations in SIV-related technology. I suggest integrating this information into a new paragraph in your introduction so that the reader is more aware of the possibilities regarding ethical and safety issues that are reasonable to suspect could be helpful or harmful.
- Line 156: please cite.
- Concept section: Similarly to my suggestion above, your description of prevention versus response strategies in this section is very helpful and would be important to address in your introduction more thoroughly to justify the focus on these types of studies.
- Lines 185-186: It would read better to reword the information inside the parentheses as a separate sentence.
- Lines 206-207. Please justify more fully why you will not “conduct a critical appraisal, assess methodological limitations, or evaluate the risk of bias due to the nature of study.” Every study would likely benefit from these practices, so please include more rationale for these choices. Furthermore, this statement is confusing as just after this (e.g., line 211), there is a statement regarding bias mitigation. Therefore, it would be best to either have a subsection describing such bias mitigation strategies, methodological limitations, etc., and/or more fully explain what is meant by the statement in lines 206-207.
- Line 212: you write that you will conduct a pilot and refinement stage, followed by the sentence, “The research team will test it on at least three studies to confirm its accuracy and completeness.” I believe that this sentence is your description of what your piloting stage will entail, but this is slightly unclear. Please connect that this is your piloting plan, if that is correct.
- Data Analysis section: please include a citation regarding your intended qualitative data analysis, and name your specific qualitative analytic approach (I believe you are describing thematic analysis). I also suggest expanding more on your plan to use reflexivity, including what the process will entail and why it is recommended and by whom.
- Line 232: please cite.
