# Supplementary material for: Using safe and ethical technology to prevent and respond to sexual and interpersonal violence during adolescence and young adulthood: Identifying evidence, best practices, and pathways forward—A global scoping review protocol
Source: PLoS One. 2025 Aug 13;20(8):e0320709. doi: 10.1371/journal.pone.0320709 (PMC12349042; doi:10.1371/journal.pone.0320709)
Supplement: Attachment — Submitted filename: Response to Reviewers 7.15.docx [file pone.0320709.s005.docx]

**RESPONSE MATRIX**

**15 July 2025**

**Manuscript ID: PONE-D-25-09607**

**Manuscript Title: Using Safe and Ethical Technology to Prevent and Respond to Sexual and Interpersonal Violence during Adolescence and Young Adulthood: Identifying Evidence, Best Practices, and Pathways Forward—A Global Scoping Review Protocol**

| **Reviewers’ Comments (#1)** | **Authors’ Responses (Page and line number)** |
| --- | --- |
| 1. Summary (Abstract)  • The abstract provides a concise overview of the rationale, objectives, and methods.  • To further improve clarity, consider specifying the types of studies included (e.g., qualitative, quantitative, mixed-methods) directly in the summary. This will help readers quickly understand the scope and methodological breadth of your review. | Thank you for your suggestion. We clarified the inclusion of qualitative, quantitative, and mixed-methods studies during the review. The following sentence was added: “We will include qualitative, quantitative, and mixed-methods studies.”(Abstract, lines 37–38) |
| 2. Background and Study Objectives • The background is comprehensive and well-referenced, clearly establishing the need for this review. • To strengthen this section, explicitly reference any previous systematic or scoping reviews on similar topics and clarify how your review specifically addresses the existing gaps identified in previous literature. For example, detail the unique aspects of safety and ethical considerations that have not been systematically synthesized to date (aspects like data protection regulations, user consent, or risk management strategies could add depth). | We cited relevant existing reviews and emphasized how our review uniquely addresses a gap in the literature. The following sentence was added “Our study fills this gap by mapping how these safety and ethical elements are integrated into technology-based interventions targeting SIV among adolescents and young adults.” (Background section, lines 135–137) |
| 3. Clearly delineating primary and secondary objectives would enhance the reader’s understanding of your review’s focus areas and priorities. | We reworded the objectives to clarify that the primary aim is to assess safety and privacy integration in SIV-related technologies, while secondary aims include exploring practical implementation, technology types, target populations, and future research needs. (Background, lines 144–152) |
| 4. Methods  • The methods section is generally robust, with clear descriptions of the search strategy, screening, and data extraction processes.  • Specify exclusion criteria more explicitly (e.g., non-English studies, non-youth populations, non-technology interventions).  • Additionally, provide a more detailed justification for the selected timeframe (2008 to present), explaining its relevance to the evolution of technology in this field.  • Clarify how grey literature or unpublished studies will be handled, as this can impact the comprehensiveness of your review. | We added explicit exclusion criteria, including non-English publications, non-youth populations, non-technology-based interventions, and non-original research. (Exclusion Criteria section, lines 199–204)  We clarified that the 2008 start date reflects the emergence of smartphones and widespread use of digital tools relevant to SIV interventions. The following sentences was added “reflecting the rapid evolution of digital technologies following the launch of the first smartphones. This timeframe captures the rise of mobile and internet-based tools that have transformed how SIV is addressed. (Context section, lines 195-198)  We clarified our approach to grey literature by explicitly listing key sources (e.g., UN Women, USAID, OAIster) and noting their value in identifying reports and materials beyond peer-reviewed journals. (Search Strategy, lines 217–220) |
| 5. Supplementary Materials • The inclusion of a detailed search strategy and extraction form is a notable strength, supporting reproducibility and transparency | Thank you for highlighting this. |
| 6. Limitations  • The protocol acknowledges the limitation of including only English-language studies, which may introduce language bias. This is appropriate, but you may also wish to discuss any other potential limitations, such as the challenges in synthesizing a highly heterogeneous evidence base. | We expanded the Limitations section to include potential challenges related to synthesizing a highly heterogeneous evidence base. The following sentence was added “Additionally, the expected heterogeneity in study designs, populations, technologies used, and outcomes measured may pose challenges for synthesis and may limit the comparability and generalizability of findings.” (Limitations section, lines 313–315) |
| 7.In summary, your protocol is methodologically sound and addresses a critical area of public health. Addressing the points above will further enhance its clarity, transparency, and alignment with best practices for scoping reviews. I look forward to seeing the results of your important work. | Thank you for this and other helpful critiques which have strengthened our manuscript. |
| **Reviewers’ Comments (#2)** | **Authors’ Responses** |
| 1.Thank you for this important forward thinking innovation to the field! Consider revising the title & short title to include 'youth' or 'young people' as an important descriptor. | We revised the full and short titles to include “during Adolescence and Young Adulthood.” (Title Page, Lines 2 & 4,5) |
| 2.Review grammar in abstract Obj sentence | We corrected the grammar from “addresses” to “address.” (Abstract, line 30) |
| 3.Consider revising the terms used. Interpersonal violence is not typically considered a sub-category of GBV as stated in line 55. Rather, interpersonal violence includes a wider range of behaviors including community (gang, etc) violence. I think the term implies a bigger scope than is envisioned here. You might mean intimate partner violence? See <https://www.ncbi.nlm.nih.gov/books/NBK525208/>. Consider aligning with a recent terminology pub: <https://ecpat.org/wp-content/uploads/2025/04/Second-Edition-Terminology-Guidelines-final.pdf> | Thank you for the suggestion. We retained the original terminology "sexual and interpersonal violence (SIV)" to maintain consistency with our research framing. However, we revised the Background to clarify our conceptualization of SIV as a major category of gender-based violence (GBV), encompassing sexual assault, dating violence, and intimate partner violence. (Background, lines 57–60) |
| 4.Please add definitions of terms such as 'online grooming', 'revenge pornography', and 'sextortion' | Brief definitions were added in parentheses to clarify each term. “Online grooming (manipulating minors for sexual purposes), revenge pornography (non-consensual sharing of intimate images), and sextortion (threats to release sexual content for more material)” (Background, lines 87–89) |
| 5.Please include the full list of search terms | We added more search terms in the main text and explicitly referenced the full list in Supplementary File S2. “The search, conducted in January 2025, incorporated a wide array of keywords related to SIV, such as “intimate partner violence,” “gender-based violence,” “interpersonal violence,” “sexual abuse,” “sexual assault,” “sexual harassment,” and “femicide.” Technology-related terms included “mobile app,” “mobile applications,” “smartphone apps,” “portable software application,” “eHealth,” “digital health,” “telemedicine,” and “technology-facilitated abuse.” The full list of search terms and database-specific queries is provided in Supplementary File S2.” (Search Strategy, lines 210-215) |
| 6.Did you consider including online research on experiences of SV/GBV? It could be an important addition for the field. | Thank you for the suggestion and we agree with you. Currently, we have chosen not to include online research studies that focus solely on participants' experiences of SV/GBV outside the context of specific technological interventions. However, this is an important area of work and was noted in the discussion section of the paper. |
| 7.Please add a citation for the sentence: "a pivotal age range when SIV rates are at their highest before decreasing." | We added a citation to support this sentence in the revised manuscript. (Inclusion Criteria, Line 180) |
| 8.Consider expanding the resources for grey/white literature to include ECPAT, WeProtect Global Alliance, and other tech-focused organizations. | Thank you for the helpful suggestion. As our search was completed after submitting the study protocol, we are unable to expand the sources further. We add this as one limitation. The following sentence was added “Our search strategy may have missed relevant grey literature from key organizations such as ECPAT and the WeProtect Global Alliance, as the search was finalized prior to protocol submission.” (Limitation, Line 135-137) |
| 9.Data extraction should clearly document the type of violence addressed, type of data (if any) collected (personally identifiable information? violence outcomes? indirect outcomes? no outcomes at all?) | We revised the Data Extraction section to specify analysis methods, technology type, violence type, and data collected. (Data Extraction, lines 250–256) |
| 10.What do you mean by 'facilitators' to be extracted? | We clarified the meaning of “facilitators” as “features or conditions that enabled effective implementation.” (Data Extraction, lines 253-254) |
| 11.In the discussion, you talk about informing city/state governments. Since this is a global study, can 'national' governments also be added? | We revised the text to include "national" governments alongside city and state levels. (Discussion, line 294) |
| **Reviewers’ Comments (#3)** | **Authors’ Responses** |
| 1.Please use consistent either APA or AMA-style in-text citation formatting. AMA would include superscript numbering, while APA would include the author last names and publication date in parentheses. | We updated all in-text citations and references to follow AMA style consistently throughout the manuscript. |
| 2.General recommendation: please clarify more thoroughly, in both the introduction and method, why a scoping review is the best choice of review type for this study. | We expanded the rationale for selecting a scoping review by explaining its suitability for mapping diverse and emerging literature, particularly in a field where concepts, technologies, and ethical considerations are still evolving. (Background, line 137; Methods, lines 158–162) |
| 3.Abstract:  Please include the full version of the abbreviation PRISMA-P. | We added the full version of the abbreviation “PRISMA-P” as “Preferred Reporting Items for Systematic Reviews and Meta-Analyses Protocols”. (Abstract, Line 40) |
| 4.Briefly mention that Covidence is a type of software for those who are unfamiliar | We added a brief clarification that Covidence is “a web-based platform for systematic review management” to assist readers unfamiliar with the tool. (Abstract, lines 41-42) |
| 5.I have an issue with the way SIV is presented in the first paragraph. Think about and revisit. | We revised the first paragraph to more clearly define SIV as a subset of GBV. The following sentence was added “One major category of GBV is sexual and interpersonal violence (SIV), which includes a range of behaviors such as sexual assault (e.g., non-consensual sexual contact), dating violence (e.g., controlling or abusive behavior in romantic relationships), and intimate partner violence (e.g., physical or emotional abuse by a current or former partner).” (Background, lines 57-60) |
| 6.Line 67: “age-groups” does not need a hyphen. | We revised the phrase “age-groups” to “age group” to correct the grammar. (Background, line 71) |
| 7.Line 70: “(11,12).Sexual”: insert a space. | We inserted a space between the citation and the word “Sexual” for proper formatting. (Background, line 74) |
| 8.Line 80: “Adolescents and young adults are easy target of “: change to targets. | We changed “target” to “targets” for grammatical accuracy. (Background, line 84) |
| 9.Line 81: “both in partner and non-partner contexts”: please change to “partnered and non-partnered” | We revised the phrase to “partnered and non-partnered” for improved clarity. (Background, line 85) |
| 10.Line 82: the word “penetration” is inappropriately used in this sentence. Do you mean frequency or prevalence? | We revised “penetration” to “prevalence” to more accurately reflect the intended meaning. (Background, line 86) |
| 11.Line 85: please say “in the United States” | We revised the sentence to specify “in the United States”. (Background, lines 90) |
| 12.Line 85: “Youth aged 13-17 years were found to be at the highest risk of technology-assited abuse, with majority of the perpetrators being older than this age-group (18).” Perhaps a more precise and grammatically correct way to word this is something like: In fact, one study found that youth aged 13-17 were the age group with the highest risk of being victimized by technology-assisted abuse, with the majority of perpetrators being older than this age group (18). | We revised the sentence for clarity and grammatical accuracy using the suggested phrasing. (Background, lines 90-92) |
| 13.Line 94: “use(21,22)” insert a space after “use.” | We inserted a space after "use" to correct the formatting. (Background, line 99) |
| 14.Lines 94-98: Please be more specific about the “topics” that adolescents may develop “incorrect” perceptions about; also, please be more specific about what is meant by “content-based data practices” and survivor-centered “design.” I also suggest citing all of these claims. | We revised the section to specify that adolescents may develop incorrect perceptions of consent, victim-blaming, and healthy relationships. We clarified “survivor-centered design” with examples such as anonymized reporting and trauma-informed interfaces and added appropriate citations. (Background, lines 101-104) |
| 15.Line 103: What is an example of a potential ethical issue that may have been reported in the past? | We added an example of ethical concerns. The following sentence was added “ For example, some platforms have lacked sufficient anonymization or security, resulting in the unintentional disclosure of survivor identities.” (Background, lines 122-124) |
| 16.Lines 113-120: please be sure to cite all claims. | We reviewed and added citations to support all claims made in this section. (Background, lines 131-143) |
| 17.Method:  General recommendation: please include a description of how the “needs of diverse populations” will be methodologically approached, such as whether it will be a specific research question or piece of extracted data, as this goal is mentioned in both the introduction and discussion. | We added “population characteristics (e.g., age, gender identity, sexual orientation) ”to the Data Extraction section (Data Extraction, lines 247-248) |
| 18.Line 135: add period. | Period added at the end of the sentence for proper punctuation. (Method, line 139) |
| 19.Research question 2: This is a great question, yet it might be more scientifically fruitful to answer if it is reworded as an open question. For example, “To what extent has technology been effective in facilitating…” may give you more room to answer this question qualitatively. | We revised Research Question 2 to be more open-ended: “To what extent has technology been effective in facilitating incident reporting, enhancing survivor safety, and improving access to support services?” (Review Questions, line 168-169) |
| 20.The parenthetical portion of your 3rd research question is important background information about ethical considerations in SIV-related technology. I suggest integrating this information into a new paragraph in your introduction so that the reader is more aware of the possibilities regarding ethical and safety issues that are reasonable to suspect could be helpful or harmful. | We moved the content regarding ethical and safety considerations from the third research question into a dedicated paragraph in the Background section to provide better context and clarity. (Background, lines 114–118) |
| 21.Line 156: please cite. | We have added an appropriate citation to support the statement. (Inclusion Criteria, line 180) |
| 22.Concept section: Similarly to my suggestion above, your description of prevention versus response strategies in this section is very helpful and would be important to address in your introduction more thoroughly to justify the focus on these types of studies. | We added a more detailed explanation of prevention versus response strategies in the Background section to provide clearer justification for focusing on these study types. The distinction is also briefly mentioned again in the Concept section to maintain alignment. (Background, lines 107–113) |
| 23.Lines 185-186: It would read better to reword the information inside the parentheses as a separate sentence. | We reworded as “PubMed/MEDLINE” (Search Strategy, lines 216) |
| 24.Lines 206-207. Please justify more fully why you will not “conduct a critical appraisal, assess methodological limitations, or evaluate the risk of bias due to the nature of study.” Every study would likely benefit from these practices, so please include more rationale for these choices. Furthermore, this statement is confusing as just after this (e.g., line 211), there is a statement regarding bias mitigation. Therefore, it would be best to either have a subsection describing such bias mitigation strategies, methodological limitations, etc., and/or more fully explain what is meant by the statement in lines 206-207. | We clarified that “we will not conduct a critical appraisal, assess methodological limitations, or evaluate the risk of bias, as the purpose of a scoping review is to provide a broad overview of existing literature rather than evaluate the quality of individual studies.” To address concerns about bias mitigation, we expanded the data analysis section to detail how reflexivity, memo-writing, and team discussions will support transparency and credibility. (Evidence Screening and Selection, lines 236-239; lines 264-268) |
| 25.Line 212: you write that you will conduct a pilot and refinement stage, followed by the sentence, “The research team will test it on at least three studies to confirm its accuracy and completeness.” I believe that this sentence is your description of what your piloting stage will entail, but this is slightly unclear. Please connect that this is your piloting plan, if that is correct. | We revised the sentence to “The form will undergo a piloting stage, during which two reviewers (MS and SB) will test it on at least three studies to ensure accuracy, completeness, and consistency.”(Data Extraction, lines 243–245) |
| 26.Data Analysis section:  please include a citation regarding your intended qualitative data analysis and name your specific qualitative analytic approach (I believe you are describing thematic analysis). I also suggest expanding more on your plan to use reflexivity, including what the process will entail and why it is recommended and by whom. | We have identified reflexive content analysis as the analytic approach and cited related references to support its appropriateness for scoping reviews. We also elaborated on our reflexivity strategy, including memo-writing and team discussions, and explained how these practices support analytical rigor. (Data Analysis, lines 264-268) |
| 27.Line 232: please cite. | We have added an appropriate citation to support the statement. (Deviations from the protocol, lines 273) |
| **Reviewers’ Comments (#4)** | **Authors’ Responses** |
| 1.I recommend for the authors to outline how they intend to share their findings from the study once it is completed. | We added a new section titled “Dissemination” to describe our plan to share findings with peers. (New section after Discussion, lines 299-305) |
